# Demonstration of Interoperability Between MIDRC and N3C: A COVID-19 Severity Prediction Use Case

**DOI:** 10.1007/s10278-025-01605-4

**Published:** 2025-08-14

**Authors:** Heather M. Whitney, Rachel Baccile, Hui Li, Karen Drukker, Christopher Meyer, Nicholas P. Gruszauskas, Weijie Chen, Diane S. Lauderdale, Sandy Napel, Seyed Kahaki, Rui Carlos Sá, Chris Beesley, Brandy Phalora, Sam Michael, Robert L. Grossman, Ken Gersing, Maryellen L. Giger, and on Behalf of the National COVID Cohort Collaborative (N3C) Consortium, Adam B. Wilcox, Adam M. Lee, Alexis Graves, Alfred Anzalone, Amin Manna, Amit Saha, Amy Olex, Andrea Zhou, Andrew E. Williams, Andrew Southerland, Andrew T. Girvin, Anita Walden, Anjali A. Sharathkumar, Benjamin Amor, Benjamin Bates, Brian Hendricks, Brijesh Patel, Caleb Alexander, Carolyn Bramante, Cavin Ward-Caviness, Charisse MadlockBrown, Christine Suver, Christopher Chute, Christopher Dillon, Chunlei Wu, Clare Schmitt, Cliff Takemoto, Dan Housman, Davera Gabriel, David A. Eichmann, Diego Mazzotti, Don Brown, Eilis Boudreau, Elaine Hill, Emily Carlson Marti, Emily R. Pfaff, Evan French, Farrukh M. Koraishy, Federico Mariona, Fred Prior, George Sokos, Greg Martin, Harold Lehmann, Heidi Spratt, Hemalkumar Mehta, J. W. Awori Hayanga, Jami Pincavitch, Jaylyn Clark, Jeremy Richard Harper, Jessica Islam, Jin Ge, Joel Gagnier, Johanna Loomba, John Buse, Jomol Mathew, Joni L. Rutter, Julie A. McMurry, Justin Guinney, Justin Starren, Karen Crowley, Katie Rebecca Bradwell, Kellie M. Walters, Ken Wilkins, Kenneth R. Gersing, Kenrick Dwain Cato, Kimberly Murray, Kristin Kostka, Lavance Northington, Lee Allan Pyles, Lesley Cottrell, Lili Portilla, Mariam Deacy, Mark M. Bissell, Marshall Clark, Mary Emmett, Matvey B. Palchuk, Melissa A. Haendel, Meredith Adams, Meredith Temple-O’Connor, Michael G. Kurilla, Michele Morris, Nasia Safdar, Nicole Garbarini, Noha Sharafeldin, Ofer Sadan, Patricia A. Francis, Penny Wung Burgoon, Philip R. O. Payne, Randeep Jawa, Rebecca Erwin-Cohen, Rena Patel, Richard A. Moffitt, Richard L. Zhu, Rishi Kamaleswaran, Robert Hurley, Robert T. Miller, Saiju Pyarajan, Sam G. Michael, Samuel Bozzette, Sandeep Mallipattu, Satyanarayana Vedula, Scott Chapman, Shawn T. O’Neil, Soko Setoguchi, Stephanie S. Hong, Steve Johnson, Tellen D. Bennett, Tiffany Callahan, Umit Topaloglu, Valery Gordon, Vignesh Subbian, Warren A. Kibbe, Wenndy Hernandez, Will Beasley, Will Cooper, William Hillegass, Xiaohan Tanner Zhang

**Affiliations:** 1 Department of Radiology, University of Chicago, Chicago, IL USA; 2 Medical Imaging and Data Resource Center, Chicago, IL USA; 3 Institute for Translational Medicine, Chicago, IL USA; 4 Center for Translational Data Science, University of Chicago, Chicago, IL USA; 5 Division of Imaging, Diagnostics, and Software Reliability, Office of Science and Engineering Laboratories, CDRH, US FDA, Silver Spring, MD USA; 6 Department of Public Health Sciences, University of Chicago, Chicago, IL USA; 7 Department of Radiology, Stanford University School of Medicine, Stanford, CA USA; 8 National Institute of Biomedical Imaging and Bioengineering, National Institutes of Health, Bethesda, MD USA; 9 Regenstreif Institute, Indianapolis, IN USA; 10 Office of the Director, National Center for Advancing Translational Sciences, National Institutes of Health, Bethesda, MD USA

**Keywords:** Interoperability, COVID-19, Comorbidity, Multi-modality

## Abstract

Interoperability between data sources, one of the FAIR (Findability, Accessibility, Interoperability, and Reusability) principles for scientific data management, can enable multi-modality research. The purpose of our study was to investigate the potential for interoperability between an imaging resource, the Medical Imaging and Data Resource Center (MIDRC), and a clinical record resource, the National COVID Cohort Collaborative (N3C). The use case was the prediction of COVID-19 severity, defined as evidence for invasive ventilatory support, extracorporeal membrane oxygenation, death, or discharge to hospice in the N3C clinical record. Patient-level matching between MIDRC and N3C was identified using Privacy Preserving Record Linking via an honest broker. We identified positive COVID-19 tests and chest radiograph procedures in N3C and used the interval between them to identify images with matching intervals in MIDRC. Of the 236 patients (306 unique images) meeting initial inclusion criteria in MIDRC, 117 patients (and 139 unique images) remained after date interval matching between repositories and exclusion of patients with multiple potential matches. The Charlson Comorbidity Index (CCI) and the minimum mean arterial pressure (MAP) on the day of the chest radiograph were used as clinical indicators. The AUC in the task of predicting severe COVID-19 was evaluated using the computer-extracted imaging index alone (MIDRC), clinical indicators alone (N3C), and both together. Our model combining imaging and clinical indicators (CCI over 2 and MAP below 70) to predict severe COVID had an AUC of 0.73 (95% CI 0.62–0.84), and the models including imaging or clinical indicators alone were 0.67 (95% CI 0.56–0.79) and 0.69 (95% CI 0.59–0.80), respectively. This study highlights the potential for cross-platform data sharing to facilitate future multi-modality research and broader collaborative studies.

## Introduction

Data commons and repositories are an important resource for big-data analysis that can lead to advances in medicine. They have the potential to contribute multi-modal data, bringing together data derived from medical images and from clinical records. The National Institutes of Health (NIH) launched several data initiatives in response to the coronavirus-19 (COVID-19) pandemic, including the Medical Imaging and Data Resource Center (MIDRC) [1] and the National COVID Cohort Collaborative (N3C) [2]. These massive data resources have the potential to support retrospective evaluation of data that can then serve as use cases for future medical advancements, including use of artificial intelligence and machine learning methods [3].

Interoperability between data resources can be particularly high impact for medicine [4, 5] and is part of the FAIR [6] principles for data (Findability, Accessibility, Interoperability, and Reusability). In the context of FAIR, interoperability is defined as “the ability of data or tools from non-cooperating resources to integrate or work together with minimal effort” [6]. Governance models, which oversee data ingestion, storage, maintenance, and disposal, have a substantial influence on interoperability [5]. Challenges for data interoperability for COVID-19 research were identified early on in the pandemic [7] and addressing them has been an ongoing area of effort, for purposes such as integrating genomic and clinical data [8] and vaccine response data [9]. A recent review highlighted the lack of interoperable datasets for COVID-19, especially for imaging-related data [10].

**Fig. 1:**
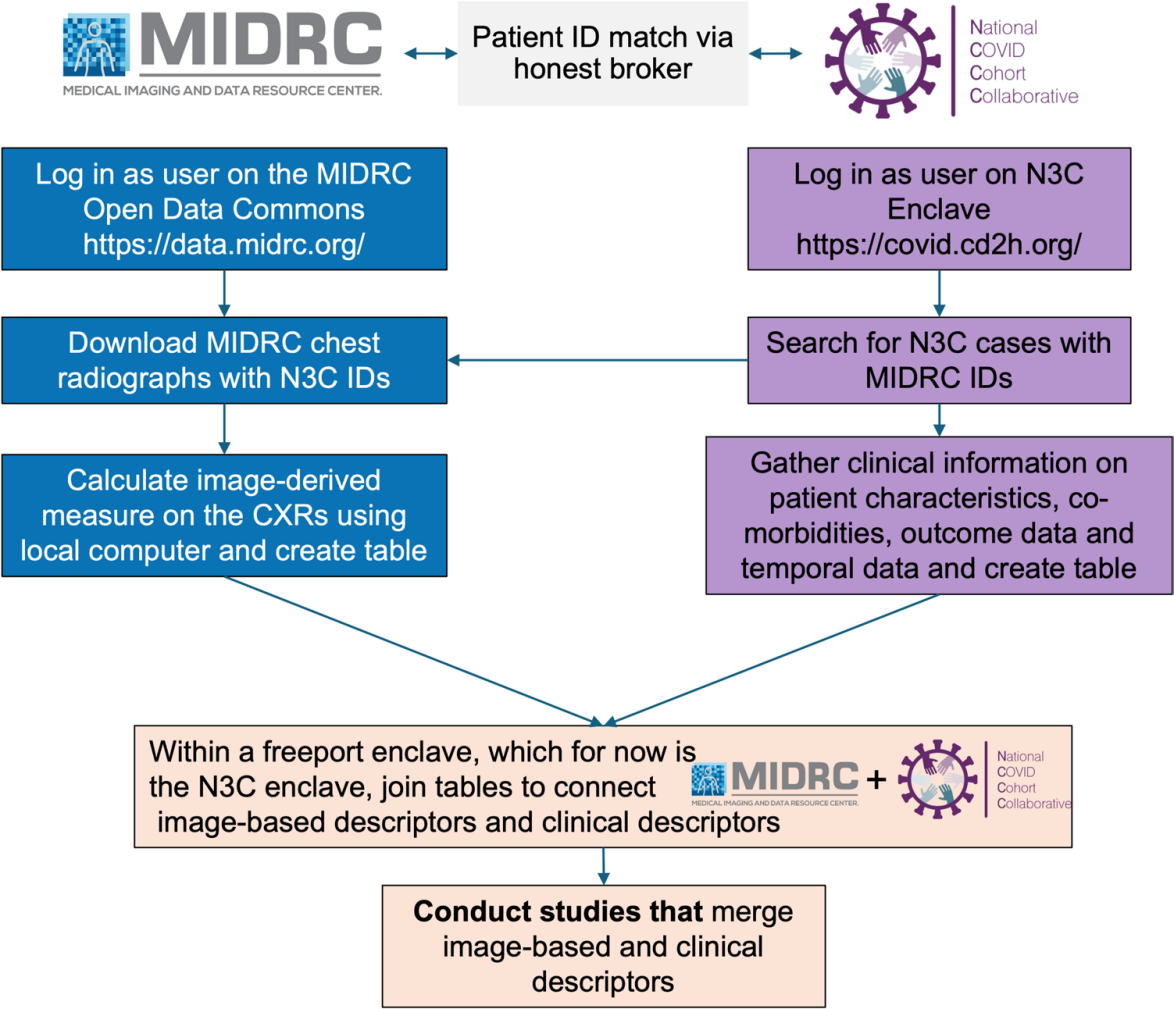
Conceptual overview of interoperability between MIDRC and N3C

MIDRC and N3C operate under two different data privacy governance models. Images in the open MIDRC data commons are fully de-identified during the ingestion process. The MIDRC de-identification process includes randomly shifting dates while maintaining the relative sequence of events for each patient, ensuring the integrity of their longitudinal timeline. This means that only* relative* data with respect to imaging exam dates are available, such as the interval between imaging acquisition and an associated measurement (such as a COVID-19 test). On the other hand, data in N3C exist as a Limited Dataset [11, 12], which means that actual dates are associated with patients (e.g., dates of imaging procedures and dates of laboratory tests). Integrating imaging data from MIDRC and clinical data from N3C requires coordination and collaboration between the two data resources via an honest broker [13] to accurately match patients, align dates, and achieve interoperability capabilities that may enhance the use of each dataset (Fig. 1).

The purpose of our study was to demonstrate the interoperability between MIDRC and N3C via a use case of predicting severe outcomes for COVID-19 patients. We describe the logistics needed for the interoperability use case as well as example models. Three measures of predicting severe outcomes were investigated: (a) a previously-published computer-extracted measure using imaging alone (using images from MIDRC), (b) a measure derived from the clinical record alone (using data from N3C), and (c) a measure that combined data from both (i.e., imaging + clinical data).

## Materials and Methods

### Study Population

Privacy-preserving software services were established between MIDRC, N3C, and the honest broker at Regenstrief Institute [14]. Regenstrief acted as the honest broker for both MIDRC and N3C. When a MIDRC Globally Unique Identifier (“MIDRC ID”), which is privacy preserving, was passed to Regenstrief via a MIDRC Gen3 data mesh service (gen3.org), Regenstrief, as the honest broker, could perform a match to see if there was matching data within N3C. If N3C passed an N3C Identifier (“N3C ID”) to Regenstrief, Regenstrief could perform a match and return privacy-preserving MIDRC IDs to N3C. Due to the sensitivity of the data and the agreements used to collect the data, in all cases, N3C data (including N3C patient IDs) never left its secure enclave. The matched and integrated data was always analyzed within the N3C secure enclave, which is approved to manage controlled access data and authorized to interoperate with MIDRC [15]. With these services, a study population of subjects with data in both MIDRC (identified with a MIDRC ID) and N3C (identified with a N3C ID) was created. For these patients, the following steps were taken.

### Imaging Index from Images in MIDRC

Unique de-identified patient MIDRC IDs were downloaded from N3C via a data download request. These MIDRC IDs were used to query the MIDRC open data commons for imaging studies relevant for this work according to two stages of inclusion criteria: (a) chest radiographs that were acquired no later than 7 days from a positive COVID-19 test result (“C19 + test result”) and (b) acquired in anterior–posterior view. A computer-extracted imaging-derived measure of COVID-19 severity was determined for each chest radiograph using a previously published deep learning model [16] that was developed to predict the development of severe COVID-19 within 24 h of the C19 + test result. The model uses a sequential learning framework that was successively trained in four phases; full technical details can be reviewed at the original publication [16]. In that study, severe COVID-19 was defined as patient admission to intensive care and/or evidence of intubation from the clinical record. Using the existing algorithm, the “imaging index” was generated for each eligible chest radiograph. Note that in the present study, severe COVID-19 was defined differently, as described below. Thus, the imaging index served as a surrogate measure of severe COVID-19 for this study. Note that because the focus of this study is to demonstrate interoperability and an existing algorithm was used, this study did not develop, train, or validate a new imaging-based algorithm to predict COVID-19 severity.

### Matching of the Specific Imaging Exam (MIDRC) and Specific Clinical Procedures (N3C)

To match data between MIDRC and N3C, the time interval between a patient’s positive C19 + test and a chest radiograph procedure in N3C was calculated and matched exactly to the MIDRC interval. Patients were excluded if there was no exact matching date interval, either due to missing C19 + test results, missing a documented chest radiograph procedure, or the time interval between the two in N3C did not match the time interval in MIDRC. Because some patients had multiple C19 + tests and chest radiograph procedures recorded in N3C, the patient cohort was separated into subcohorts based upon the number of imaging studies available in MIDRC, the number of intervals between a C19 + test and imaging studies in MIDRC, and the number of matching intervals between a C19 + test and image procedure in N3C (Table 1). Patients with one imaging study, one interval between C19 + test and imaging study in MIDRC, and one matching interval within N3C were used in the final cohort.

**Table 1:** Subcohorts identified through combinations of imaging studies in MIDRC and number of intervals between COVID-19 tests in MIDRC and in N3C. Patients in Subcohort 1 were used in the final analysis of this study. C19 +, COVID-19 positive

Sub cohort	Number of imaging studies in MIDRC	Number of intervals between C19 + test and imaging studies in MIDRC	Number of intervals between C19 + test and image procedure in N3C
1	One imaging study	One interval	One matching interval
2	One imaging study	One interval	One matching interval on multiple calendar days
3	One imaging study	Multiple intervals	Multiple matching intervals

### Clinical Indicators from Data in N3C

Within N3C, the Charlson Comorbidity Index (CCI) [17, 18] at the admission date of the associated hospitalization and the minimum mean arterial pressure (MAP) on the day of the chest radiograph recorded in N3C were used as the clinical indicators in modeling. These indicators were identified for their use in other studies of COVID-19 outcomes using data from N3C [19–22]. The CCI was determined using the CCI Logic Liaison template available within the N3C enclave. For each chest radiograph, the patient’s CCI was classified into two categories: (a) CCI score between 0 and 2 and (b) CCI score 3 or greater. The minimum MAP was classified as either low (< 70) or not low (≥ 70) [23].

### Predictive Models for Severe COVID-19

Three predictive models for severe COVID-19 were evaluated for their potential to demonstrate interoperability between MIDRC and N3C: (a) using the radiographic AI-predictive model (imaging index alone), (b) using clinical indicators alone, and (c) using the combination of the imaging index and clinical indicators. In the present study, severe COVID-19 was defined as evidence for invasive ventilatory support, extracorporeal membrane oxygenation, death, or discharge to hospice [22] included within the clinical record held at N3C via the invasive respiratory support Logic Liaison template available within the N3C enclave. A logistic regression model was used to predict severe COVID-19, and standard errors were calculated at the patient level to account for multiple images within one imaging study (i.e., clustered data).

### Statistical Analysis

The odds ratio for the factors in each logistic model were determined, along with their 95% confidence intervals (CI) using standard errors for clustered data to account for multiple images in one imaging exam for some patients. Specifically, we applied the Huber-White (HC0) sandwich estimator with clustering at the individual level to obtain standard errors using the Imtest R package [24]. The imaging index ranges from 0 to 1 and was represented in the model as tenths (0.1 units), so that the odds ratio represents the difference in odds of severe COVID-19 associated with a 0.1 unit change in the index. Receiver operating characteristic (ROC) analysis [25] was conducted in the task of predicting severe COVID-19. The area under the ROC curve (AUC) and 95% CIs were determined empirically using the R pROC package [26] for each of the three predictive models and served as the figure of merit.

## Results

### Study Population Characteristics

**Fig. 2:**
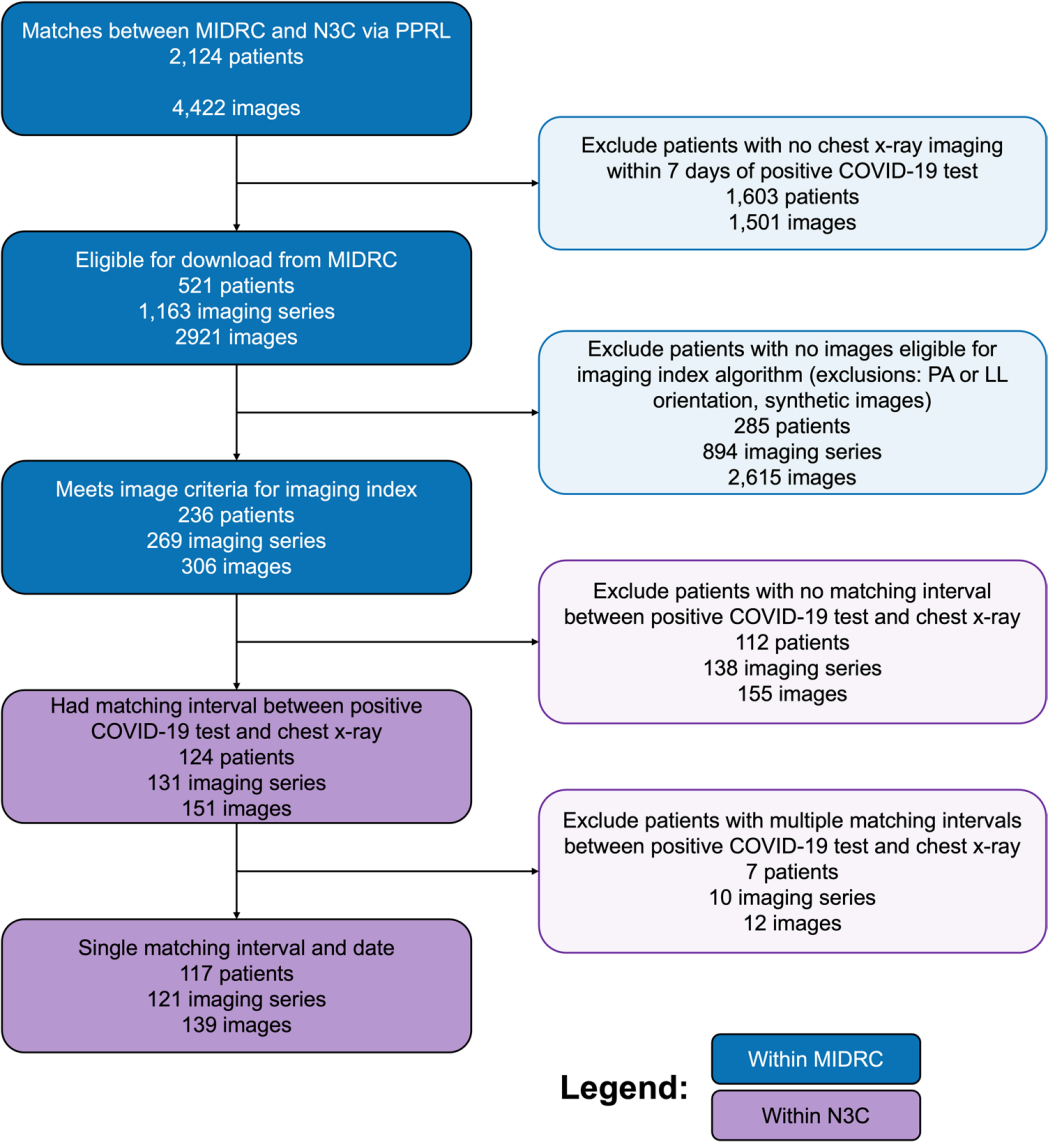
Consort figure of patients included in the study

The final data set included 139 unique images from 121 imaging studies of 117 patients that had chest radiographs between June 3, 2020, and April 24, 2023. The consort figure for the inclusion criteria in the study is shown in Fig. 2.

Of the 139 images, 22 (15.83%) were associated with severe COVID, 62 (44.60%) had a CCI of 3 or greater, and the median minimum MAP was 78.30 [IQR 69.17, 89.75] (Table 2). The cohort was primarily Black (105, 75.54%) and male (78, 56.12%) and had a median age of 60 [IQR 47.50, 74].

**Table 2:** Patient characteristics in the cohort used in this study (by image)

Characteristic	*N* (%) or median [IQR]
Demographics	
Race/ethnicity	
Black not Hispanic	105 (75.54)
Hispanic or Latino	< 20
White not Hispanic or Latino	< 20
Asian not Hispanic or Latino	< 20
Other race not Hispanic or Latino	< 20
Sex	
Male	78 (56.12)
Female	61 (43.88)
Age, median [IQR]	60 [47.5, 74]
Clinical indicators	
CCI category	
0–2	77 (55.40)
3 +	62 (44.60)
Low MAP (< 70)	36 (25.90)
Imaging indicator	
Image index, median [IQR]	0.24 [0.04, 0.62]
Outcomes	
Severe COVID-19	22 (15.83)

Severe COVID-19 was identified for patients who had *any* clinical indicator

### Statistical Analysis

In the model predicting severe COVID-19 using only the imaging index, there was an estimated odds ratio of 1.19 per tenth imaging unit (95% CI 1.00–1.42), indicating a marginal association with greater odds of severe COVID-10. In the model using only clinical indicators (CCI over 2 and MAP below 70), the odds ratio for severe COVID-19 was 4.94 (95% CI 0.95–25.56) for a high CCI and 0.61 (95% CI 0.19–1.98) for low MAP. When the imaging index and clinical indicators were combined, odds ratios for all factors remained similar, though none reached statistical significance at the 0.05 level. However, these results should be interpreted with caution, as the study is underpowered to detect significant associations. Regression results are shown in Table 3.

**Table 3:** Regression results for the prediction of severe COVID-19 using the imaging index alone and the clinical indicators. CCI, Charlson comorbidity index; MAP, mean arterial pressure; OR, odds ratio; CI, confidence interval

	Model 1: Imaging index alone		Model 2: Clinical indicators alone		Model 3: imaging index + clinical indicators	
	OR	95% CI	OR	95% CI	OR	95% CI
Imaging index	1.19	(1.00, 1.42)	-	-	1.12	(0.95, 1.32)
CCI: 3 +	-	-	4.94	(0.95, 25.56)	3.89	(0.77, 19.73)
Minimum MAP: < 70	-	-	0.61	(0.19, 1.98)	0.61	(0.18, 2.02)

The regression factors for the imaging index were multiplied by 10 to support interpretability, such that the OR is associated with an increase in the imaging index of 0.1

**Fig. 3:**
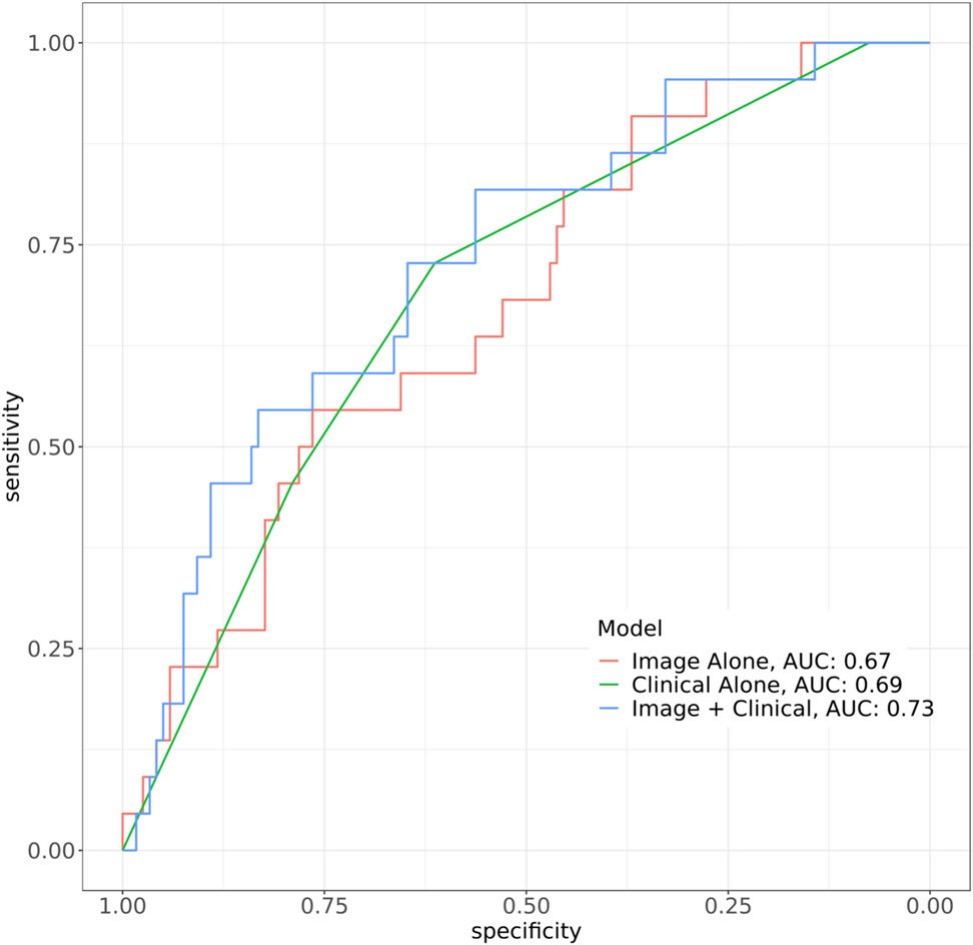
Receiver operating characteristic curves in the task of predicting severe COVID-19 from imaging index alone, clinical indicators alone, or the combination of imaging index and clinical indicators. AUC, area under the receiver operating characteristic curve

The AUC in the task of predicting severe COVID-19 using the imaging index alone was 0.67 (95% CI 0.56–0.79), and using clinical indicators (CCI over 2 and MAP below 70) alone was 0.69 (95% CI 0.59–0.80). The model combining imaging and clinical indicators to predict severe COVID had a greater median AUC (0.73, 95% CI 0.62–0.84) than the models including imaging or clinical indicators alone. ROC curves for the three models are shown in Fig. 3.

## Discussion

Interoperability between different data repositories allows matching different types of data available for the same patient and plays a critical role in realizing the great potential of multimodal data analysis to improve healthcare. In particular, data commons interoperability enables curation of multimodal datasets that are foundational for the development of data-fusion AI, which has emerged as a promising technology to combine multiple types of data (e.g., radiology, pathology, clinical lab measurements, genomics) in various clinical tasks. In this study, interoperability between MIDRC (imaging data) and N3C (clinical data) has been demonstrated for the use case of prediction of severe COVID-19 using three predictive models. Two of them used the data from each repository alone and one used it in combination, the latter specifically facilitated by interoperability coordination efforts. The results demonstrate the potential for future discovery and hypothesis-driven studies, such as optimizing clinical data used for the task and subcohort analysis, or using other summary measures of comorbidity (such as the Elixhauser [27, 28] or Quan-Charlson indices [29]).

Limitations of this study included that the interoperability demonstration incorporated somewhat ad hoc choices for the intervals between C19 + test and imaging and for the clinical data windows used. During the development of the study, we reviewed the data and saw negligible change in, e.g., the CCI if the window was different. However, other studies may be more sensitive and may need to rely on more strict definitions of intervals. We also limited the study to straightforward matches in interval between imaging and clinical variables in order to simplify the demonstration of interoperability. In the future, more improvements are needed for series-based matches (as opposed to image-based matches, which was the focus here). The interval check was not optimal but was what was possible now because of date shifting (including potentially unknown date shifting between two commons). This limitation impacted the size of the final dataset, which limited the power to detect statistical significance. Expansion of interoperability will allow for research on larger cohorts by increasing the confidence in the matching of imaging data in MIDRC to clinical data in N3C.

The comparison of models was also limited in terms of statistical power due to the small sample size. Nonetheless, there was evidence of effects worth exploring with a larger case series. Different interoperability procedures are needed to expand the dataset to additional patients and to extend the analysis to other subcohorts. Thus, this work shows the potential for interoperability and future hypothesis-driven work, including at specific operating points.

## Conclusion

Interoperability between MIDRC and N3C has been demonstrated for the first time via the use case of predicting severe COVID-19 by combining both imaging-derived measures and data from the clinical record. Future work will investigate the incorporation of other clinical indicators and use tasks as well as an in-depth evaluation of how integrating imaging and clinical data improves clinical decision-making in this task and other tasks.

## Data Availability

The imaging data are freely available at https://data.midrc.org. The clinical data of the N3C dataset were available to the authors by Data Use Request DUR-BB40587 and are not available to the public. Code for processing the N3C dataset is not available due to restrictions on the Limited Dataset. In general, all code shareable by MIDRC is available at https://github.com/MIDRC.
